# Intestinal Alkaline Phosphatase Combined with Voluntary Physical Activity Alleviates Experimental Colitis in Obese Mice. Involvement of Oxidative Stress, Myokines, Adipokines and Proinflammatory Biomarkers

**DOI:** 10.3390/antiox10020240

**Published:** 2021-02-04

**Authors:** Aleksandra Danielak, Dagmara Wojcik, Agnieszka Mazur-Bialy, Marcin Surmiak, Jan Bilski, Aneta Targosz, Marcin Magierowski, Anna Chmura, Malgorzata Strzalka, Gracjana Krzysiek-Maczka, Katarzyna Magierowska, Urszula Szczyrk, Sławomir Kwiecien, Agata Ptak-Belowska, Tomasz Brzozowski

**Affiliations:** 1 Department of Physiology, Faculty of Medicine, Jagiellonian University Medical College, 16 Grzegorzecka Street, 31-531 Cracow, Poland; aleksandradanielak26@gmail.com (A.D.); dagmara1.wojcik@uj.edu.pl (D.W.); marcin.surmiak@uj.edu.pl (M.S.); aneta.targosz@uj.edu.pl (A.T.); m.magierowski@uj.edu.pl (M.M.); anna.1.chmura@uj.edu.pl (A.C.); malgorzata.strzalka@uj.edu.pl (M.S.); gracjana98@gmail.com (G.K.-M.); katarzyna.magierowska@uj.edu.pl (K.M.); urszula.szczyrk@uj.edu.pl (U.S.); skwiecien@cm-uj.krakow.pl (S.K.); agata.ptak-belowska@uj.edu.pl (A.P.-B.); 2 Department of Biomechanics and Kinesiology, Faculty of Health Sciences, Jagiellonian University Medical College, 20 Grzegorzecka Street, 31-531 Cracow, Poland; agnieszka.mazur@uj.edu.pl (A.M.-B.); jan.bilski@uj.edu.pl (J.B.)

**Keywords:** experimental colitis, intestinal alkaline phosphatase, exercise, inflammatory bowel disease, physical activity, oxidative stress, myokines, adipokines, antioxidative factors

## Abstract

Intestinal alkaline phosphatase (IAP) is an essential mucosal defense factor involved in the process of maintenance of gut homeostasis. We determined the effect of moderate exercise (voluntary wheel running) with or without treatment with IAP on the course of experimental murine 2,4,6-trinitrobenzenesulfonic acid (TNBS) colitis by assessing disease activity index (DAI), colonic blood flow (CBF), plasma myokine irisin levels and the colonic and adipose tissue expression of proinflammatory cytokines, markers of oxidative stress (SOD2, GPx) and adipokines in mice fed a standard diet (SD) or high-fat diet (HFD). Macroscopic and microscopic colitis in sedentary SD mice was accompanied by a significant decrease in CBF, and a significant increase in the colonic expression of tumor necrosis factor-alpha (TNF-α), IL-6, IL-1β and leptin mRNAs and decrease in the mRNA expression of adiponectin. These effects were aggravated in sedentary HFD mice but reduced in exercising animals, potentiated by concomitant treatment with IAP, especially in obese mice. Exercising HFD mice demonstrated a substantial increase in the mRNA for adiponectin and a decrease in mRNA leptin expression in intestinal mucosa and mesenteric fat as compared to sedentary animals. The expression of SOD2 and GPx mRNAs was significantly decreased in adipose tissue in HFD mice, but these effects were reversed in exercising mice with IAP administration. Our study shows for the first time that the combination of voluntary exercise and oral IAP treatment synergistically favored healing of intestinal inflammation, strengthened the antioxidant defense and ameliorated the course of experimental colitis; thus, IAP may represent a novel adjuvant therapy to alleviate inflammatory bowel disease (IBD) in humans.

## 1. Introduction

Inflammatory bowel disease (IBD) comprises two major phenotypic types: Crohn’s disease (CD) and ulcerative colitis (UC), both characterized by chronic course with relapsing and remitting phase [1]. In most patients, ulcerative colitis is confined to the colon and rectum, whereas Crohn’s disease can occur in any part of the gastrointestinal tract. Although the precise etiology of IBDs remains unknown, it has been revealed that the combination of genetic and environmental factors promotes epithelial barrier dysfunction, which in turn enables the gut microbiome to penetrate intestinal mucosa, leading to the disturbance of the mucosal immune system and subsequent chronic inflammation [2,3,4]. The changes in gut microbiota, considered as a crucial environmental factor in IBD, are thought to result from novel diet patterns, which is consistent with the observation that the prevalence of IBD is increasing steadily in Western countries and newly industrializing countries, possibly because of the westernization of lifestyle associated with higher consumption of processed food, fat and refined sugar as well as with physical inactivity [5].

Abnormal hypertrophy of mesenteric white adipose tissue (mWAT) is recognized as a characteristic feature of Crohn’s disease and leads to fat wrapping, described as the expansion of fat from the mesentery to partially cover the small and large intestines [6]. Accumulating evidence suggests that the hypertrophied mWAT is implicated in the pathogenesis of IBD and actively contributes to the elevated expression of proinflammatory cytokines as well as to the disturbance in adipokines secretion [7,8,9,10]. Interestingly, the analysis of CD severity among obese patients revealed that obesity was positively correlated with increased activity of disease and a higher number of hospitalizations [11]. An important tool to understand the changes associated with westernized lifestyle is an established model of diet-induced obesity (DIO), which imitates the features of obesity observed in humans, induces gut inflammation and exacerbates experimental colitis [9,12,13,14].

On the other hand, recent findings seem to indicate that the effects of proinflammatory stimuli may be counteracted by anti-inflammatory action of physical activity mediated to some extent by proteins released from exercising skeletal muscles, the so-called “myokines”, such as irisin involved in the browning of white adipose tissue [15,16,17,18,19]. The emerging role of skeletal muscle as an endocrine organ [20], which produces various agents capable of modulating visceral fat metabolism and reversing the effects of adipokines, constitutes an important step towards understanding the crosstalk between skeletal muscle and adipose tissue. Furthermore, physical activity has been postulated as an adjunctive therapy in CD [21], and several studies have shown an improvement in the quality of life and stress level in patients with inactive or mildly active CD following a moderate-intensity exercise program [22,23]. This seems to be in keeping with the beneficial influence of voluntary, but not forced, exercise observed in animal studies, which have revealed the alleviation of experimental colitis and intestinal inflammation in response to physical exercise [24,25].

Intestinal alkaline phosphatase (IAP) has been recently recognized as an important mucosal defense factor necessary for maintaining gut homeostasis [4,26,27]. IAP belongs to the superfamily of alkaline phosphatase (AP) responsible for hydrolyzing a variety of monophosphate esters and is secreted both to the intestinal lumen and to the bloodstream [28,29]. IAP is not only a crucial regulator of the intestinal mucosal permeability but also plays an essential role in promoting mucosal tolerance to the gut microbiome due to, at least partly, a dephosphorylation of bacterial LPS [4,30]. Exogenous IAP has been shown to ensure protection against intestinal and systemic inflammation in a variety of diseases, such as necrotizing enterocolitis, antibiotic associated diarrhea and metabolic syndrome, and a potential therapeutic role of IAP in IBD has also been suggested [27]. In a human trial, the administration of IAP daily over a 7 day course to patients with Crohn’s disease resulted in a short-term improvement in disease activity scores [31]. However, whether combined therapeutic strategies, namely, the application of voluntary exercise and IAP administration, could potentiate the mechanism of protection against the development of colitis has not been studied yet.

Therefore, the aim of our study was to determine whether IAP combined with voluntary physical activity could affect experimental colitis induced by intrarectal administration of 2,4,6-trinitrobenzenesulfonic acid (TNBS) in mice fed a standard diet (SD) or high-fat diet (HFD). We attempted to examine if a HFD exacerbates the course of colitis in mice as demonstrated before [9,10] and whether this effect could be prevented to some extent by voluntary physical activity combined with IAP treatment, adding more evidence on the existence of possible muscle–fat crosstalk. Additionally, we aimed to investigate the underlying molecular mechanisms involved in the beneficial action of the combination of IAP and voluntary exercise, namely, the alterations in disease activity index (DAI), colonic blood flow (CBF), irisin release, as well as the colonic expression of proinflammatory biomarkers, myokines (irisin), adipokines and antioxidizing enzymes. We also aimed to determine for the first time whether the effect of IAP treatment combined with voluntary exercise could exhibit additional benefits in the mechanism of exercise-induced protection against experimental colitis and attenuation of intestinal inflammation.

## 2. Materials and Methods

### 2.1. Animals and Diets

A pathogen free room with a 12 h/12 h light cycle was selected for housing male C57BL/6 mice. These mice had unrestricted access to water and food and were adapted to being kept in the animal room for at least two weeks after purchase. The study designed two types of feeding for the animals for a total of 12 weeks. First, diet C 1090-70, based on fat in the percentage of 42% to cover 70% energy and considered as a high fat diet (HFD), was applied. Second, diet C 1000 consisting of the regular chow diet served as the control to HFD (C 1090-70) and is considered as standard feeding for lean mice (SD, standard diet). Both diets were purchased from Altromin Company (Lage, Germany). The cholesterol concentration was 200 mg/kg in the high fat diet C 1090-70, while the energy delivered by this diet reached the value of 5495.855 kcal/kg. In contrast, the energy value for the C 1000 diet was 3518.05 kcal/kg. Diet C 1090-70 included energy metabolism from fat (3784 kcal/kg), carbohydrates (771 kcal/kg) and protein (829 kcal/kg). In the case of the control diet C 1000, the share of organic ingredients in covering metabolic energy requirements was as follows: fat (457 kcal/kg), protein (691 kcal/kg) and carbohydrates (2358 kcal/kg). Since soybean phytoestrogens may contribute to the interpretation of data obtained in animals fed HFD compared to animals fed a control diet, the control diet (C 1000), according to the information provided from manufacturer, did not contain these compounds. Detailed fatty ingredients for both SD and HFD were as follows: the C 1000 diet (SD) [mg/kg]: arachidic acid C-20:0 50; eicosanoic acid C-20:1 150; alpha-linolenic acid C-18:3 150; linolenic acid C-18:2 28,500; palmitic acid C-16:0 2500; stearic acid C-18:0 1350, and oleic acid C-18:1 13,500 and for C 1090-70 diet (HFD) [mg/kg]: arachidic acid C-20:0 1226; eicosanoic acid C-20:1 680; alpha-linolenic acid C-18:3 7155; linolenic acid C-18:2 54,103; palmitic acid C-16:0 46,668; stearic acid C-18:0 27,259, and oleic acid C-18:1 34,724, respectively. The study was granted permission from the local Ethics Committee of the Jagiellonian University Medical College in Cracow, Poland (I Local Ethical Committee No. 19/2016, KRA1_19_2016), and the experimental protocol was carried out in accordance with the Helsinki Declaration.

### 2.2. Experimental Design

After the adaptation period, SD- and HFD-fed mice were randomly assigned into seven experimental series each consisting from 6–10 animals per group: (1) sedentary mice fed SD, (2) mice kept on SD and subjected to voluntary physical activity, (3) mice fed SD administered intragastrically i.g. with IAP (200 U/day) and subjected to voluntary exercise, (4) sedentary mice kept on HFD, (5) sedentary mice fed HFD and administered i.g. with IAP (200 U/day), (6) mice fed HFD and subjected to voluntary exercise, (7) mice kept on HFD administered i.g. with IAP (200 U/day) and allowed to exercise in running wheels.

Four groups of animals fed SD or HFD for 12 weeks were subjected to voluntary wheel running (Activity Wheel and Living Chamber; Lafayette Instrument Company, IN, United States) to evaluate the influence of physical activity on the course of experimental colitis. Other remaining groups were considered as sedentary animals. During the experiment, the sedentary and exercising mice still received either the C 1000 or C 1090-70 obesity diets. The voluntary-exercising mice were kept in cages equipped with a running wheel assembly connected to a device that counted rotations of the wheel, which were recorded daily for six weeks of exercise, and transmitted the data on the frequency and running rate to a USB hub, so that they could be detected by software dedicated to data storage and analysis for variable time periods.

After 6 weeks of wheel running, the exercise sessions were stopped and three groups of animals fed either SD or HFD were administered oral IAP (200 U/day) (Sigma Aldrich St. Louis, MO, USA, cat # P0114), which was mixed with drinking water and provided to the mice ad libitum. The remaining groups received an equal volume of drinking water without IAP. The experimental colitis was induced by intracolonic administration of 2,4,6-trinitrobenzenesulfonic acid (TNBS) in both sedentary and exercising mice after 2 weeks of IAP treatment, as described elsewhere [32]. During the period of the study, the daily measurement of the energy intake (kilocalories per day) together with weekly determination of body weight was performed.

### 2.3. Induction of Colitis

The animals were anesthetized with isoflurane and experimental colitis in both groups of mice: SD and HFD were induced by intracolonic administration of TNBS (Sigma, Slough, UK) at a dose of 4 mg per mouse in 50% ethanol. For this purpose, a 200 μL volume dissolved in a 50% solution of ethanol or an equal volume of a 0.9% saline solution was introduced intrarectally. The mice in the control group were administered 50% ethanol at a volume of 200 μL per mouse, in accordance with the volume administered to the mice with TNBS.

The animals were housed separately after the induction of colitis and had their food intake and body weight monitored daily. The animals were weighed and anesthetized at day 4 post-colitis induction, and subsequently, the abdominal cavity was opened. After the separation of the colon, the CBF was determined in the areas not affected by inflammatory lesions using the laser Doppler flowmetry technique. Mice were euthanized, and the disease activity index (DAI) was determined in accordance with a modification of previously published clinical score [32]. Briefly, the severity of the colonic disease DAI includes scoring for diarrhea and lethargy (0–3) and rectal bleeding evaluation comprising a visual inspection of blood in feces and the perianal area (0–4) [9,32].

The inferior vena cava was slightly dissected, and a venous blood sample was collected from this blood vessel to evaluate the plasma levels of irisin. For this purpose, blood was collected in sterile plastic EDTA vials and left on ice during further centrifugation. The blood samples were then centrifuged at 1000× *g* for 10 min at 15 °C, and the plasma was stored at −80 °C. The EIA kit (Kit No. EK-067-16, Phoenix Pharmaceuticals Inc., Burlingame, CA, USA) was used to determine the content of irisin in the blood. Within-test variability and interstudy variability were <10% and <15%, respectively.

The adipose tissue from the abdominal mesenteric region was collected and transferred to tubes, snap frozen in liquid nitrogen and stored at −80°C until RNA extraction analysis [33].

### 2.4. Determination of TNF-α, IL-6, IL-1β, Adiponectin, Leptin, SOD2 and GPx Transcripts by Quantitative Real-Time Polymerase Chain Reaction (PCR) Assay and Reverse Transcriptase Polymerase Chain Reaction (RT-PCR)

Assessment of gene expression in colonic mucosa and mesenteric adipose tissue samples was performed by quantitative real-time polymerase chain reaction (qRT-PCR). The total RNA from both colonic mucosa and adipose tissue was extracted using an RNeasy Plus Mini Kit (Qiagen, Hilden, Germany). The NanoDrop 2000 spectrophotometer (Thermo Scientific, Wilmington, DE, USA) was used to evaluate the concentration and quality of RNA. RNA was reverse-transcribed using a High-Capacity RNA-to-cDNA Kit (Applied Biosystems, Foster City, CA, USA). Real-time polymerase chain reaction (PCR) was performed using a StepOne Plus system (Applied Biosystems, Foster City, CA, USA) and applying a TaqMan Gene Expression Master Mix (Applied Biosystems) and TaqMan Gene Expression Assays (Applied Biosystems) for TNF-α, IL-6, adiponectin, leptin, SOD2 and GPx. The β-actin was used as a housekeeping gene. The sequences of the primers are available following request. The relative gene expression was calculated according to a 2^−ΔΔ*C*t^ method. In some tests, RT-PCR was also performed for better visualization in the unchanged colon mucosa of intact rats or those with TNBS colitis fed with different diets SD and HFD with or without IAP administration to assess the alteration in the colonic mRNA expression of adiponectin, leptin, SOD2 and GPx mRNA. For RT-PCR assessment, the colonic mucosal samples weighing approximately 200 mg were scraped using a glass slide. Thereafter, mucosal scrapings were immediately quick-frozen in liquid nitrogen and stored at −80 °C until RT-PCR analysis. The method of Chomczynski and Sacchi was used to isolate total RNA from mucosal samples [34]. The concentration and quality of the extracted RNA was measured on a spectrophotometer. Then, 5 µg of total mRNA was reverse transcribed into cDNA using StrataScript reverse transcriptase and oligo (dT) primers (Stratagene) in a DNA thermocycler (Perkin-Elmer-Cetus, Norwalk, CT, USA). RT-PCR analysis included the following primer nucleotide sequences:

β-actin (size of PCR product 764 bp), forward: 5′-TTG TAA CCA ACT GGG ACG ATA TGG-3′, reverse: 5′-GAT CTT GAT CTT CAT GGT GCT AGG-3′ (accession nos V01217, J00691), adiponectin (size of PCR product 282 bp), forward: 5′-GATGGCAGAGATGGCACTCC-3′(accession XM_032899235.1), reverse: 5′-CTTGCCAGTGCTGCCGTCAT-3′; leptin (size of PCR product 142 bp), forward: 5′-GAGTAGAGTGAGGCTTCCAGGA-3′(accession KX255819), reverse: 5′-TGCTGCAGATAGCCAATGAC-3′, SOD2 (size of PCR product 241 bp), forward: 5′-GCACATTAACGCGCAGATCA-3′, reverse: 5′-AGCCTCCAGCAACTCTCCTT-3′ (accession XM_032894729), GPx (size of PCR product: 197 bp), forward: 5′-CCTCCAGTACGTCCGACCTG-3′, reverse: 5′-CAATGTCGTTGCGGCACACC-3′ (accession XM 032910620).

### 2.5. Statistical Analysis

Results are expressed as means ± S.E.M. The data were processed by statistical analysis software SPSS version 16.0 (SPSS Inc., Chicago, IL, USA). Statistical significance between 2 groups or more than 2 groups was calculated using a Student’s t-test or a two-way ANOVA test with Tukey’s post hoc test, respectively. Differences of *p* < 0.05 were considered statistically significant.

## 3. Results

### 3.1. Effect of Voluntary Exercise and IAP Administration on DAI, Colonic Blood Flow, Histology of Colonic Mucosa and Plasma Irisin Level in Mice Fed SD or HFD

**Figure 1:**
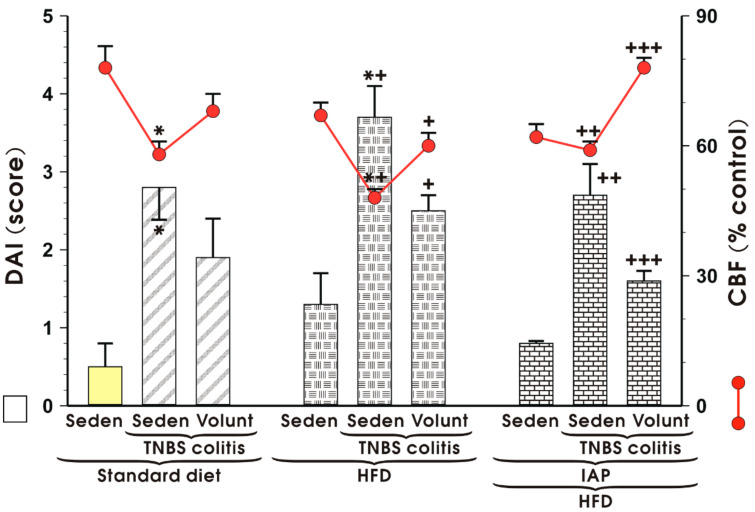
Effect of voluntary exercise with or without treatment with intestinal alkaline phosphatase (IAP) on the disease activity index (DAI) and the accompanying alterations in colonic blood flow (CBF), presented as red lines in this figure, in sedentary (Seden) mice with 2,4,6-trinitrobenzenesulfonic acid (TNBS)-induced colitis fed standard diet (SD) or high-fat diet (HFD). Results are mean ± S.E.M. of eight animals per group, * significantly different (*p* < 0.05) from sedentary mice fed SD, *^+^ significantly different (*p* < 0.05) from sedentary mice fed HFD, ^+^ significantly different (*p* < 0.05) from animals fed SD not subjected to voluntary exercise, ^++^ significantly different from sedentary mice fed HFD with colitis (*p* < 0.05) without IAP administration, ^+++^ significantly different (*p* < 0.05) from sedentary mice fed HFD with or without IAP administration.

Figure 1 shows the alterations in DAI score measured in the colonic mucosa as well as the changes in CBF of sedentary or exercising mice fed either an SD or a HFD with or without IAP administration. In the sedentary TNBS colitis mice fed a HFD, a significant increase in DAI and a significant decrease in CBF were observed compared to that measured in the sedentary group with colitis kept on SD (*p* < 0.05) (Figure 1). These effects obtained in the HFD mice were reversed when animals were subjected to voluntary exercise, as documented by a substantial reduction in DAI accompanied by a significant increase in CBF compared with non-exercising mice fed a HFD (*p* < 0.05) (Figure 1). The combined treatment of IAP and voluntary physical activity on wheel running in the HFD mice resulted in a further significant decrease in DAI along with a significant elevation in CBF as compared with the respective values in exercising HFD mice with or without IAP administration (*p* < 0.05) (Figure 1).

**Figure 2:**
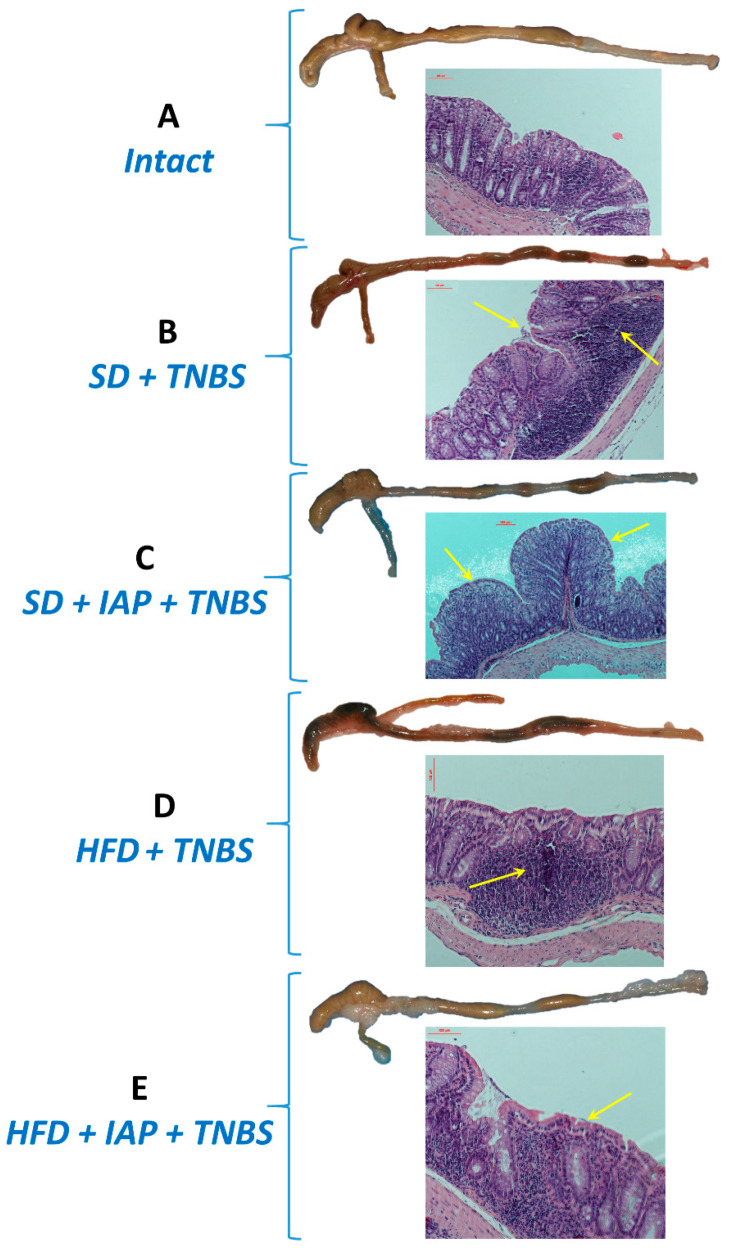
The representative gross (upper panel) and microscopic (lower panel) appearance of colon from intact mouse (**A**), sedentary mouse with TNBS colitis fed standard diet (SD) (**B**), sedentary mouse fed SD with colitis treated with IAP with voluntary exercise on wheels (**C**), sedentary mouse fed HFD with colitis (**D**) and mouse fed HFD with colitis treated with IAP with voluntary exercise on wheels (**E**). In mice fed SD or HFD with colitis, the enterocyte architecture is distorted, revealing edema and infiltration of mucosa and submucosa with inflammatory cells, destroyed crypts, extended ulcerations and transmural inflammatory cell infiltrates (**B**,**D**) (arrows) as compared with exercising mice fed either with SD or HFD treated with IAP (**C**,**E**). Treatment with IAP, especially in HFD exercising mice, led to partial restoration of colonic mucosal lining (arrows), partial preservation of enterocyte structure, low levels of epithelial damage and less severe inflammatory cell infiltration of the colonic mucosa and submucosal structure.

Figure 2A–E show the alterations in the gross appearance and histology of the intact mouse colon and those observed in mice with colitis fed SD or HFD subjected to physical activity and administered with or without IAP. The macroscopic appearance of the intact healthy colon (Figure 2A) and the appearance of the colon in non-exercise control mice kept on SD and treated or untreated with IAP are shown in Figure 2B,C, respectively. For comparison, the macroscopic and microscopic appearance of the colon in HFD-fed mice with experimental colitis treated or untreated IAP is shown in Figure 2D,E, respectively. No significant damage to the colonic mucosa was observed in healthy mice receiving SD both macroscopically and histologically. The colonic mucosa showed normal enterocyte structure, uniform thickness and no signs of inflammation (Figure 2A). On the contrary, a blood enema reflecting damage to the intestinal mucosa was observed in colitis mice kept on SD, and this effect was accompanied by focal lesions and necrosis of the colonic mucosa epithelium (Figure 2B). Less severe lesions were seen in HFD mice subjected to voluntary physical activity in conjunction with IAP administration, manifested as only superficial mucosal lesions compared to those kept on HFD without wheel running (Figure 2C vs. Figure 2B). By histology and gross inspection, the worsening of histological lesions, swelling of the colonic mucosa areas observed histologically, as well as intracolonic blood enemas were observed in sedentary mice kept on HFD. These HFD-receiving mice not subjected to physical activity presented significantly worse results of the inflammatory response compared to sedentary control mice fed an SD (Figure 2D vs. Figure 2B). All these microscopic and macroscopic alternations were reduced in mice with combined voluntary physical activity and IAP treatment as compared with sedentary HFD mice (Figure 2E vs. Figure 2D).

Table 1 shows the effect of voluntary exercise with or without IAP administration on the plasma concentrations of myokine irisin in mice fed an SD or a HFD. In the sedentary SD mice, the exposure to TNBS did not significantly affect the plasma irisin level, whereas in the sedentary HFD mice with TNBS colitis, a substantial reduction in the plasma concentration of irisin was observed as compared to that recorded in sedentary HFD animals without colitis (*p* < 0.05) (Table 1). The decrease in the plasma irisin concentration observed in the sedentary mice fed a HFD was reversed when these mice were subjected to voluntary exercise compared to the respective plasma values of this myokine in the HFD fed sedentary group (*p* < 0.05) (Table 1). Although the IAP administration tended to elevate the plasma irisin level of the exercising mice fed a HFD as compared to the group subjected to voluntary exercise but without IAP application, this effect failed to reach statistical significance (Table 1).

### 3.2. The Alterations in the mRNA Expression of Proinflammatory Biomarkers TNF-α, IL-6, IL-1β, Leptin, Adiponectin and Antioxidizing Enzymes SOD2 and GPx in Colonic Mucosa of Exercising or Sedentary Mice with or without IAP Administration

**Figure 3:**
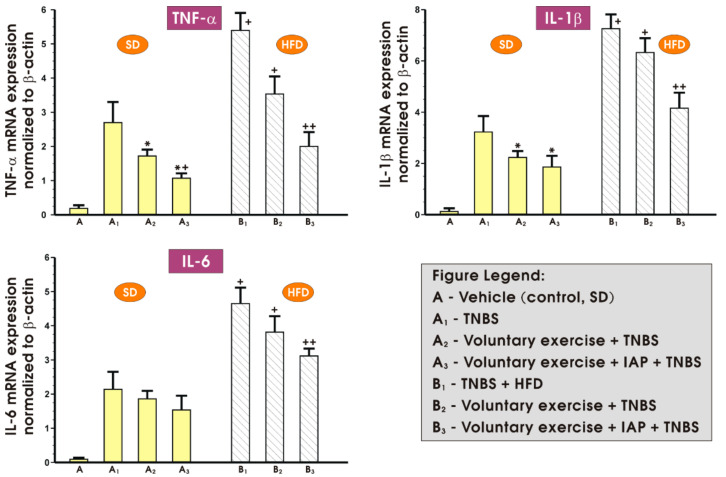
The effect of treatment with IAP combined with voluntary exercise on the mRNA expression of proinflammatory cytokines TNFα, IL-1β and IL-6 in the colonic mucosa of sedentary or exercising mice fed an SD or a HFD. Results are mean ± S.E.M. of four animals per experimental group, * significantly different (*p* < 0.05) from the sedentary colitis mice fed SD, *^+^ significantly different from (*p* < 0.05) the sedentary animals with colitis fed SD, ^+^ significantly different (*p* < 0.05) from the sedentary colitis mice fed HFD without exercise, ^++^ significantly different (*p* < 0.05) from the sedentary HFD animals with colitis subjected to voluntary exercise.

Figure 3 demonstrates the alterations in mRNA expression of proinflammatory cytokines TNF-α, IL-1β and IL-6 in the colonic mucosa of sedentary or exercising mice with colitis fed SD or a HFD with or without IAP application. In the sedentary colitis mice fed either SD or HFD, the mRNA expression of TNF-α, IL-1β and IL-6 was significantly increased compared with the respective values obtained in the group of non-exercising (sedentary) animals fed an SD (*p* < 0.05) (Figure 3). The overexpression of mRNA for TNF-α, IL-1β and IL-6 in groups fed both diets was attenuated when mice were subjected to voluntary physical activity as compared to the respective mRNA expression in sedentary animals fed SD or HFD (*p* < 0.05) (Figure 3). As for the expression of TNF-α, and IL-1β, the effect of voluntary exercise reached significance as compared to the level of expression of mRNA of these cytokines noticed in sedentary animals. Furthermore, a significant decrease in the mRNA expression of TNFα, IL-1β and IL-6 was observed, especially when voluntary physical activity was combined with IAP administration in mice fed a HFD as compared to the exercising obese mice but without IAP application (*p* < 0.05) (Figure 3).

**Figure 4:**
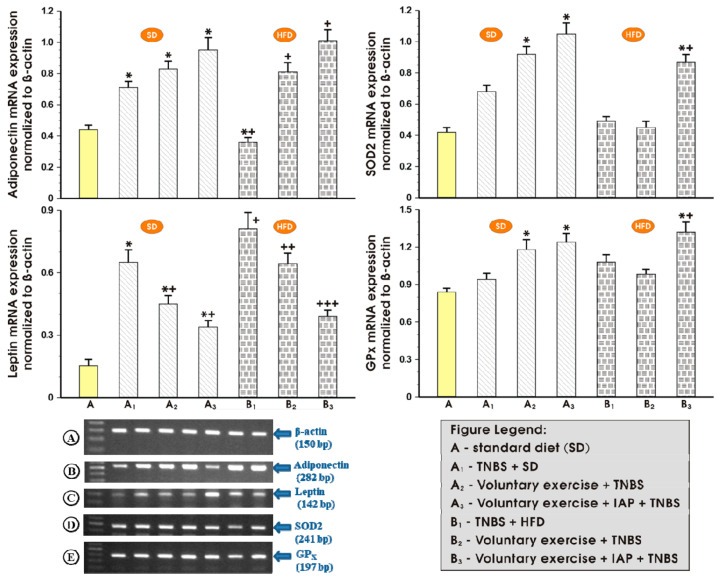
Expression of adiponectin, leptin, SOD2 and GPx mRNA determined by real-time quantitative PCR in untrained and training colitis mice with or without combination with IAP. Analysis by qualitative RT-PCR on an agarose gel of mRNA expression of β-actin (**A**), adiponectin (**B**), leptin (**C**), SOD2 (**D**) and GPx (**E**) in the colon mucosa of non-exercising or exercising mice with colonic inflammation treated or not treated with IAP. Data reflecting the mean ± S.E.M. of six animals are shown for each experimental group, * significantly different (*p* < 0.05) from the corresponding values in non-training SD-fed and colitis-free mice, *^+^ significantly different (*p* < 0.05) from the corresponding values in non-exercising colitis mice kept on SD or HFD, ^+^ significantly different (*p* < 0.05) from exercising SD or HFD fed colitis mice, ++ significantly different (*p* < 0.05) from non-exercising mice fed HFD, ^+++^ significantly different (*p* < 0.05) from exercising colitis mice kept on HFD.

Figure 4 shows the influence of treatment with IAP combined with voluntary exercise on the mRNA expression of adiponectin, leptin and antioxidizing enzymes SOD2 and GPx in the colonic mucosa of TNBS colitis mice fed an SD or a HFD. The expression of adiponectin in sedentary mice with colitis fed a HFD was significantly decreased in comparison with sedentary mice with colitis fed an SD (*p* < 0.05) (Figure 4, upper left panel). Although the physical activity exhibited a tendency to elevate the mRNA expression of adiponectin in the group fed an SD, such an effect was not significant. However, when mice fed a HFD were subjected to voluntary exercise, a significant increase in adiponectin expression was observed as compared to mice receiving a HFD without physical activity (*p* < 0.05) (Figure 4, upper left panel). Combined application of IAP administration with voluntary wheel running in mice fed a HFD further elevated the mRNA expression of adiponectin as compared with HFD mice subjected to physical exercise without IAP administration; however, this trend failed to reach statistical significance (Figure 4, upper left panel).

Figure 4 also presents the data on the leptin mRNA expression in the colonic mucosa of sedentary and exercising mice treated or not with vehicle or IAP. In the sedentary SD group of mice with TNBS-induced colitis, the overexpression of mRNA for leptin was observed as compared to the sedentary animals fed an SD without colitis (*p* < 0.05) (Figure 4, left lower panel). In the non-exercising group of mice, a HFD was associated with a significant elevation in the mRNA expression of leptin compared to the sedentary animals kept on SD, and this effect was reversed when mice fed a HFD were subjected to voluntary exercise (*p* < 0.05) (Figure 4, left lower panel). The treatment with IAP combined with concomitant voluntary physical activity significantly decreased the mRNA expression of leptin in mice fed a HFD as compared with those fed a HFD and subjected to voluntary exercise but without IAP administration (Figure 4, left lower panel).

As shown in Figure 4 (right panel), the exposure of colonic mucosa to TNBS failed to significantly affect the mRNA expression of antioxidizing enzymes SOD2 and GPx in the intestine of sedentary mice fed either SD or HFD. However, when mice with colitis fed an SD were subjected to voluntary exercise, a significant increase in the mRNA expression of SOD2 was observed compared to that measured in the sedentary SD mice (*p* < 0.05) (Figure 4, right upper panel). In contrast, physical activity failed to significantly affect the expression of GPx mRNA in mice fed either SD or HFD (Figure 4, right lower panel). Similarly to the mRNA expression of SOD2, which was markedly elevated when IAP was co-applied in mice fed a HFD subjected to physical activity (*p* < 0.05) (Figure 4, right upper panel), the expression of GPx in exercising mice fed an SD and treated with IAP was significantly increased as compared to the respective values for the mRNA expression recorded in sedentary SD mice with colitis. In HFD exercising mice, a significant increase in GPx mRNA expression was observed in exercising mice administered IAP compared to the respective values recorded in those fed HFD with colitis but without IAP administration (Figure 4, right lower panel).

### 3.3. Proinflammatory Cytokines, Leptin and Adiponectin mRNA Expression in White Adipose Tissue of Mice Subjected or not to Voluntary Exercise with or without IAP Administration

**Figure 5:**
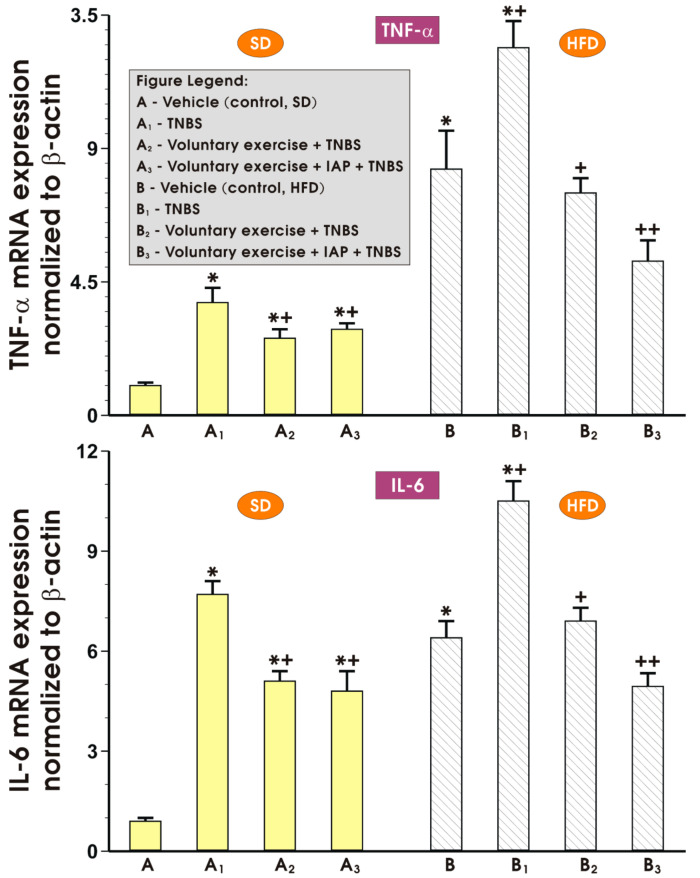
The changes in mRNA expression of TNF-α (upper panel) and IL-6 (lower panel) determined in the mesenteric fat of sedentary mice fed an SD or HFD with or without TNBS colitis and subjected or not subjected to voluntary exercise with or without treatment with IAP. Results are mean ± S.E.M. of eight animals per experimental group, * significantly different (*p* < 0.05) from the sedentary mice fed an SD, *^+^ significantly different (*p* < 0.05) from the sedentary animals with colitis fed an SD, ^+^ significantly different (*p* < 0.05) from the sedentary colitis mice fed a HFD without exercise, ^++^ significantly different (*p* < 0.05) from SD or HFD fed animals with colitis subjected to voluntary exercise.

Figure 5 shows the effect of treatment with IAP combined with voluntary exercise on the changes in mRNA expression of TNF-α and IL-6 in mesenteric white adipose tissue of the mice fed HFD or SD. In the sedentary mice fed SD or HFD, the intrarectal application of TNBS caused a substantial increase in mRNA expression of TNF-α and IL-6 in mesenteric fat (*p* < 0.05) (Figure 5, upper and lower panels, respectively). Voluntary physical activity significantly decreased the expression of these proinflammatory biomarkers in adipose tissue of mice fed SD and HFD as compared to respective values achieved in sedentary animals fed SD and HFD (Figure 5). A further increase in the expression of TNF-α and IL-6 was observed in mice fed a HFD with TNBS-induced colitis; however, when the SD and HFD mice were subjected to voluntary training, a significant decrease in the expression of TNF-α and IL-6 was observed when compared to respective values obtained in comparison with the sedentary TNBS group fed SD and HFD (*p* < 0.05) (Figure 5, upper and lower panels, respectively). The treatment with IAP combined with voluntary exercise potentiated this anti-inflammatory action, as manifested by a further significant decrease in the expression of TNF-α and IL-6 compared with animals subjected to moderate physical activity without IAP administration (Figure 5, upper and lower panels, respectively).

**Figure 6:**
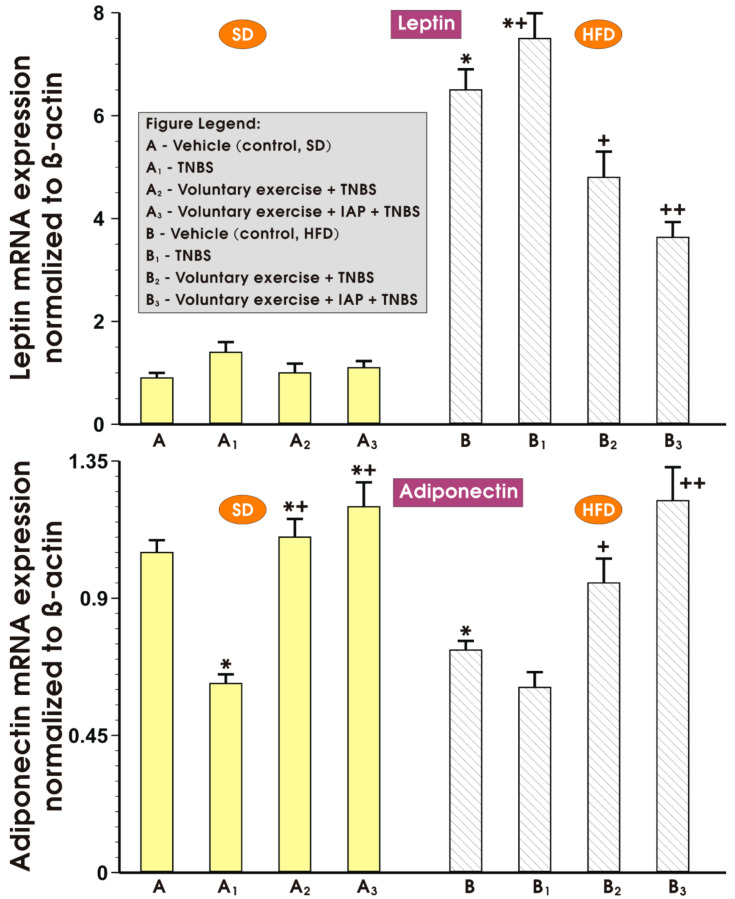
The changes in mRNA expression of leptin (upper panel) and adiponectin (lower panel) determined in the mesenteric fat of sedentary mice with or without TNBS colitis fed an SD or HFD and subjected or not subjected to voluntary exercise with or without treatment with IAP. Results are mean ± S.E.M. of eight animals per experimental group, * significantly different (*p* < 0.05) from the sedentary mice fed an SD, *^+^ significantly different (*p* < 0.05) from the animals with colitis fed SD or HFD, ^+^ significantly different (*p* < 0.05) from the sedentary mice fed an SD, ^++^ significantly different (p < 0.05) from mice fed HDF subjected to moderate exercise without IAP administration.

The alterations in mRNA expression of leptin (upper panel) and adiponectin (lower panel) determined in the mesenteric white adipose tissue of sedentary or exercising mice fed an SD or a HFD with or without IAP administration are presented in Figure 6. In the sedentary HFD mice, the expression of leptin in white adipose tissue was significantly elevated as compared with that obtained in sedentary mice fed SD (*p* < 0.05) (Figure 6). In contrast, the expression of adiponectin was significantly downregulated in sedentary mice fed HFD compared to SD mice (Figure 6, lower panel). Voluntary exercise, which failed to affect the leptin mRNA in adipose tissue of colitis mice fed SD, substantially decreased leptin mRNA expression in colitis mice fed HFD (*p* < 0.05) (Figure 6, upper panel). When the mice fed either an SD or HFD received IAP treatment combined with moderate physical activity, a further significant decrease in the expression of leptin RNA and a significant increase in the expression of adiponectin mRNA were observed compared to that measured in exercising animals fed both diets without the concomitant treatment with IAP (*p* < 0.05) (Figure 6, upper and lower panels).

## 4. Discussion

The present study confirms that HFD augmented the severity of experimental colitis as documented by the increased DAI index, the colonic tissue weight and upregulated colonic expression of proinflammatory biomarkers TNF-α, IL-1β and IL-6 mRNA compared to the mice fed an SD. These changes were accompanied by a decrease in colonic microcirculation and a reduction in the plasma levels of protective factors released by working skeletal muscle, such as irisin, together with diminished mRNA expression of anti-inflammatory adiponectin and elevated leptin expression in both colonic mucosa and white adipose tissue. However, voluntary exercise by means of wheel running reversed these inflammatory and unfavorable alterations associated with increased adiposity due to HFD via mechanisms involving the improvement in colonic blood flow, restoration of plasma irisin levels as well as attenuation of colonic expression of mRNA of proinflammatory biomarkers, to some extent confirming and further extending our previous observation in exercising mice fed diet-induced obesity [9]. Interestingly, this voluntary physical activity did influence mWAT activity, because adiponectin mRNA expression was increased, while leptin expression was reduced in the mesenteric fat tissue of exercising mice fed a HFD compared to sedentary HFD mice without physical training. Furthermore, we documented for the first time in the present study that IAP administration combined with moderate physical activity by subjecting mice to wheel running could be a novel strategy to increase the efficacy of moderate voluntary physical activity affording protection against experimental colitis via further reduction in proinflammatory biomarkers, thus accelerating the healing of colonic inflammation compared with moderate exercise alone. We observed that oral IAP potentiated the beneficial action of physical activity with respect to DAI score, which was significantly lower compared to CBF, which was substantially elevated in the exercising mice receiving IAP when compared to the group of mice subjected to moderate physical activity only. Moreover, the treatment with IAP concurrently applied in addition to moderate voluntary physical activity, especially in obese mice, elevated plasma irisin levels, considered as a protective factor [4,35,36], and increased the expression of mRNA for antioxidizing enzymes SOD-2 and GPx compared to that recorded in obese mice subjected to exercise only.

Accumulation of visceral adipose tissue and loss of muscle mass are well-documented results of physical inactivity. As a consequence of abdominal adiposity, the influx of macrophage and other inflammatory cells to the adipose tissue is triggered, leading to the promotion of inflammatory pathways [37]. Mesenteric tissue obtained from obese animals displays significantly higher macrophage infiltration compared to healthy controls [38], and it is generally accepted that increased visceral adipose tissue constitutes a pivotal source of proinflammatory factors, including TNF-α, IL-6, leptin and resistin, resulting in a low-grade chronic inflammatory state [6,38,39,40,41]. Accumulating evidence suggests that hypertrophied mWAT is among the major IBD risk factors and contributors to the pathogenesis of these disorders, which is supported by the fact that obesity can exacerbate the onset of disease and, moreover, so-called “creeping fat” clearly coincides with the extent of intestinal inflammation [42,43]. Additionally, even though CD patients are predominantly underweight, they have significantly higher ratios of intraabdominal fat to total abdominal fat compared to a control group [6,43]. As shown in the present work, HFD strongly aggravated intestinal inflammation, as reflected by significantly increased colonic indices but also by the rise in mRNA expression of TNF-α, IL-1β and IL-6 in both colonic mucosa and white adipose tissue of obese sedentary mice. These data remain in agreement with the study by Sideri et al. [44], in which a pronounced relationship between HFD and exacerbated intestinal damage in mice with TNBS colitis was demonstrated followed by the increased expression of proinflammatory biomarkers such as IL-1β, TNF-α and monocyte chemoattractant protein 1 in mesenteric adipose and intestinal tissues. Our findings are corroborative with the observation by Li et al. [45] that high-fat diet feeding exacerbated colitis in a similar model of TNBS-induced experimental colitis via induction of oxidative stress in the colonic mucosa, which may increase colonic epithelial barrier permeability and exaggerate colonic mucosal inflammation. Thus, concerning the translatory value of our work, we confirmed that diets high in saturated fats are detrimental to patients with IBD, but as shown in this experimental study, treatment with IAP and moderate physical activity deserves attention as an alternative to pharmacological therapy and a beneficial strategy to counteract these inflammatory changes associated with human IBD.

Leptin, which belongs to a family of adipokines released from white adipose tissue and is upregulated in obesity, has been shown to play an important role in the mechanism of initiation and progression of IBD exerting the proinflammatory intestinal activity due to promoting the expression of cytokines such as TNF-α and IL-6 [42,46]. The role of leptin in IBD was studied by Sitaraman et al. [46], who reported that intraluminal concentration of this adipokine was markedly elevated in patients affected by mild to severe CD compared to controls because of its overexpression in inflamed colonic epithelial cells. Similarly, we demonstrated that the expression of leptin measured in both colonic mucosa and mWAT was significantly increased in colonic mucosa during experimental colitis. Moreover, this effect was more pronounced in the mice fed a HFD than those kept on SD. In contrast to leptin, adiponectin is recognized as an anti-inflammatory factor, strongly inhibiting lipopolysaccharide-induced production of TNF-α in macrophages [10,47,48], and its expression is inversely correlated with obesity. Rodrigues et al. [49] proposed that IBD is associated with dysregulation of anti-inflammatory pathways, with the lower levels of serum and mesenteric adiponectin being of particular interest. Our data presented in this work confirmed that the adiponectin expression in the colonic mucosa and mWAT of mice fed a HFD was significantly decreased compared with the SD group of mice. Therefore, given the proinflammatory action of leptin and contradictory properties of adiponectin, it is reasonable to conclude that the downregulation of adiponectin and upregulation of leptin expression attributed to HFD could contribute to the augmented severity of the course of experimental colitis observed in our study.

Our present findings can be somehow translatory to human IBD, because Jones et al. [50] observed that IBD patients in remission with higher exercise activities were less likely to develop active disease within six months, whereas in a meta-analysis by Wang et al. [51], regular physical training was shown to decrease the risk of CD development in a group of healthy individuals. There is growing evidence pertaining to the potent anti-inflammatory properties of physical activity, with a special emphasis on the interplay between muscles and white adipose tissue, the so-called muscle–fat crosstalk. An important role of myokines, a growing family of peptides released from exercising muscles, has been suggested to underlie the anti-inflammatory action of physical activity, as these proteins are capable of counteracting noxious effects of adipokines released from white adipose tissue through their autocrine, paracrine or endocrine activities [10,20,35]. Furthermore, myokines stimulate muscle growth and hypertrophy, facilitate fat oxidation and promote insulin sensitivity [35]. One of the myokines, irisin, which is cleaved off from FNDC5 and is known to induce the browning of white adipose tissue [52], was shown in the previous work to markedly enhance wound healing in intestinal cells [53]. This observation is in keeping with our present study, which revealed that voluntary training alleviated the severity of experimental colitis in the mice fed a HFD and was positively correlated with the plasma irisin concentration. It is, therefore, possible that exercise-induced irisin could contribute to the resolution of inflammation and ultimately accelerated healing of colitis. Another attempt was made to evaluate the influence of physical activity with and without IAP treatment on mWAT endocrine activity by assessing the expression of adiponectin and leptin. We observed that the adiponectin mRNA expression in white adipose tissue of sedentary mice was downregulated, while the leptin mRNA expression was significantly increased. In the exercising obese mice treated concomitantly with IAP, the adiponectin expression was restored along with a pronounced decrease in the expression of leptin, indicating that the combination of IAP treatment with voluntary exercise might exert its beneficial effect by not yet fully recognized modifications of the muscle–fat crosstalk. A similar trend was observed in our previous studies, which revealed that mice with colitis subjected to voluntary moderate training [9] or forced moderate training [54] had significantly elevated adiponectin plasma levels, while plasma concentrations of leptin, TNF-α, IL-6 and IL-13 were markedly reduced. However, in another study, forced treadmill exercise was associated with an increase in the severity of experimental colitis, reflected by a significant elevation in DAI score and proinflammatory cytokines levels with a concomitant substantial reduction in colonic blood flow [55]. Similarly, Cook et al. [24] reported intensified severity of DSS-induced colitis, increased diarrhea incidence and mortality in mice subjected to moderate forced treadmill running. Additionally, they provided evidence that whereas forced activity, which may be perceived by animals as a chronic stressor, exacerbates intestinal inflammation, voluntary training exerts a protective effect and enhances colonic healing [24]. Discrepancies related to the conflicting outcomes of physical activity in various models may be ascribed not only to stress involvement, but also to the different intensity of exercise, which is consistent with well-documented gastro-intestinal symptoms, such as vomiting, abdominal pain and bloody diarrhea reported among athletes after ultraendurance training [56]. Jeukendrup et al. [57] concluded that LPS leakage into the bloodstream resulting in increased cytokine release could serve as an explanation for such complaints related to high intensity training. It should be noted, however, that moderate-intensity exercise appears to be safe and feasible for IBD patients in remission [23]. Moreover, in previous prospective randomized controlled trials [22,23], physical activity of different intensities failed to provoke adverse events, but it did improve the quality of life, suggesting that an improvement in the quality of patient life can be a useful additional parameter to enhance exercise benefits in human IBD.

**Figure 7:**
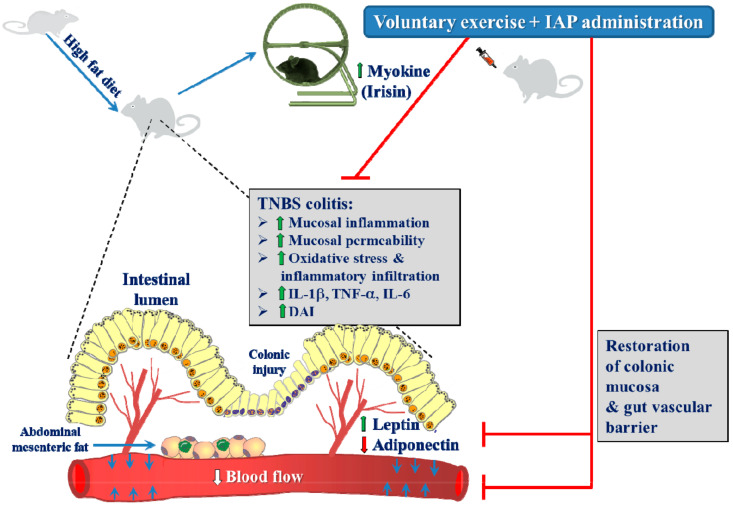
The summary scheme of the beneficial effect exhibited by the combination of IAP treatment with moderate physical activity in the mechanism of the healing of experimental colitis in mice fed HFD. The mucosal damage and an impairment in the colonic microcirculation and downregulation of antioxidative biomarkers induced by TNBS accompanied by mucosal inflammation and oxidative stress are restored by the combination of IAP and voluntary exercise due to better restoration of colonic mucosa and an improvement of gut vascular barrier caused by antioxidative myokines and adipokines.

The present study demonstrated for the first time that exogenous treatment with IAP exerted a significant protective effect on intestinal inflammation and potentiated a beneficial ameliorating effect of exercise on experimental colitis (Figure 7). Combined administration of IAP and wheel running resulted in a substantial decrease in DAI index along with the mRNA expression of proinflammatory biomarkers TNF-α, IL-1β and IL-6 in colonic mucosa, and such an effect was accompanied by a significant elevation in CBF, known to play an important role in intestinal mucosa defense mechanisms. Our data showed that the protective effects were more pronounced in the mice subjected to both physical activity and IAP application compared to the exercising animals without IAP (Figure 7). Therefore, our data are consistent with the notion that exogenous IAP ameliorates gut inflammation, enhances effect of exercise and favors healing of colitis due to enhancement in CBF and its anti-inflammatory activity. This is in keeping with the involvement of endogenous IAP in the pathogenesis of IBD, which has recently been underlined in both human and animal studies on the beneficial role of exogenous IAP in the course of human and animal IBD [31,58,59,60,61,62]. For instance, Ramasamy et al. [58] showed that IAP knockout mice presented elevated susceptibility to DSS-induced colitis, manifested by increased severity of the disease, compared to wild-type mice. They hypothesized that diminished IAP expression or activity may constitute a potential genetic factor predisposing to IBD development [55]. In another report, Tuin et al. [60] demonstrated the efficacy of oral IAP supplementation to alleviate the symptoms of IBD in a mouse model of DSS-induced chronic colitis.

The well-documented protective influence of IAP against chronic colitis may be attributed to a variety of functions, including dephosphorylation and subsequent detoxification of bacterial LPS, but also flagellin, and CpG DNA, each of them known to act as toll-like receptor (TLR) ligands [4]. As shown in previous works, LPS binds to TLR-4, leading to the activation of downstream inflammatory signaling and thus promoting proinflammatory cytokine expression [4]. However, when dephosphorylated by IAP, LPS cannot serve as a TLR-4 ligand and stimulate this pathway. Interestingly, decreased IAP mRNA expression accompanied by increased TLR4 expression resulting in IAP/TLR4 imbalance was reported in the colonic mucosa of children with IBD [63,64].

Our data showed that exogenous IAP administration in the HFD colitis mice was inversely associated with the colonic expression of proinflammatory biomarkers. Similarly, a tendency of oral IAP to decrease proinflammatory cytokine expression or plasma levels was documented in previous studies [58,60,62]. Thus, given that HFD leads to accelerated internalization of LPS from intestinal lumen and its subsequent secretion into the bloodstream resulting in endotoxemia [65,66], it is possible that IAP exhibits anti-inflammatory activity against intestinal inflammation potentiated by HFD via enhanced LPS detoxification and downregulation of its proinflammatory signaling.

Another beneficial result of LPS detoxification may be limited bacterial translocation across the mucosal barrier, which is essential in the maintenance of mucosal tolerance towards commensal microbiota [4,67]. There is growing evidence indicating that a disturbance between gut bacteria and defensive host mechanisms is implicated in the pathomechanism of IBD [68]. Olbjørn et al. [69] documented in their study that bacterial diversity was markedly reduced among IBD pediatric patients compared to healthy controls. Furthermore, the effect of IAP on gut homeostasis was studied in IAP knockout mice and revealed that mice deficient in IAP had fewer and altered types of microbes in their stools, while oral IAP administration facilitated restoration of commensal microbiota lost due to antibiotic therapy [30]. These data seem to highlight the potential therapeutic effect of IAP to treat dysbiosis and hence to heal diseases associated with dysregulated homeostasis of intestinal microbiota.

Reduced DAI score as well as diminished proinflammatory biomarker colonic expression in the presence of IAP observed in our study could also be due to compromising of the abnormal intestinal mucosal permeability. In general, the gut barrier regulated by junctional complexes, including tight junctions (TJ), prevents the excessive influx of detrimental luminal factors into the bloodstream and consequent inflammatory responses [70,71]. In line with this notion, the impaired mucosal permeability has been shown to be a crucial feature of IBD patients, but also their healthy first-degree relatives [72]. Liu et al. [70] provided evidence that IAP pretreatment preserves the localization of the components of TJ: ZO-1 and occludin and, therefore, supports intestinal barrier integrity after the addition of LPS. Based on the observation that HFD induces overexpression of proinflammatory cytokines, which in turn aggravates intestinal permeability [44], we hypothesize that IAP may reverse the detrimental alterations accompanying HFD and, therefore, strengthen the gut barrier function. However, further studies are needed to elucidate the precise mechanisms underlying IAP protective activity in the gut.

### Study Limitations

While the physiological role of FNDC5/irisin in mediating responses to exercise is unquestionable, and there is also strong evidence of altered FNDC5/irisin regulation in clinical scenarios of metabolic disorders associated with diabetes, obesity and metabolic syndrome in humans, the available methods for determining irisin are still under debate [73]. We are fully aware that there has been considerable uncertainty and concern regarding the accuracy of the measurement of serum myokine irisin from its initial introduction to the world’s basic and clinical researchers, mainly due to differences in FNDC5/irisin glycosylation [73]. For example, some studies reported unresolved problems with the nature of the transcription of the irisin precursor, type III fibronectin domain containing the gene 5 (FNDC5), in different species [73,74]. In addition, the reliability of irisin levels as measured by commercial enzyme immunosorbent assays (ELISAs) was questioned, as was the overall specificity and validity of recently published human serum reference values measured by quantitative mass spectrometry. Similar problems also occur in human protein expression samples, as some studies have not been able to show the expression of transcripts encoding truncated FNDC5 proteins by the Western blot method in humans [74]. On the other hand, the recent study of parallel measurements of irisin by ELISA and quantitative mass spectrometry [75,76] was considered credible. Despite all these discrepancies in the literature between irisin data generated in humans and rodents, determining this myokine in humans appears to be more difficult.

## 5. Conclusions

We showed that while a HFD exacerbates experimental colitis in mice, voluntary exercise ensures protection against the proinflammatory impact of obesity and improves the healing of intestinal inflammation due to the release of protective myokines, including irisin from working skeletal muscles. Moreover, our present study documented that physical activity by wheel running markedly reduced the severity of colitis as well as mRNA expression of proinflammatory biomarkers and antioxidizing enzymes in both colonic mucosa and mWAT, and these effects were potentiated by IAP administration (Figure 7). Additionally, voluntary exercise resulted in normalization of the leptin/adiponectin ratio in obese mice, together with an elevated concentration of irisin, highlighting its potent ability to modify the muscle–fat crosstalk. A combination of exercise and oral IAP results in synergistic action to ameliorate experimental colitis and favor healing of intestinal inflammation; thus it may represent a novel supportive and perhaps adjuvant therapy to alleviate IBD in humans.

## Figures and Tables

**Table 1:** The plasma levels of irisin in the sedentary mice (Seden) or those subjected to voluntary exercise (Voluntary) with or without TNBS colitis fed a standard diet (SD) or a high-fat diet (HFD) with or without concomitant treatment with IAP. Results are mean ± S.E.M. of six animals per experimental group. An asterisk indicates a significant change as compared to the respective values in the sedentary mice with or without colitis fed an SD (*p* < 0.05). An asterisk and a cross indicate a significant change as compared to the respective values in the colitis animals fed an SD (*p* < 0.05). A cross indicates a significant change as compared to the value obtained in the sedentary HFD animals with colitis (*p* < 0.05). A double cross indicates a significant change as compared to the value obtained in the sedentary HFD animals with colitis (*p* < 0.05). A triple cross indicates a significant change as compared to the value obtained in the exercising HFD animals with colitis (*p* < 0.05).

Aparance Type of Groups		Irisin (ng/mL)
*SD*	Seden	623 ± 18
	Seden + colitis	595 ± 13
	Voluntary + colitis	641 ± 16 *
	Voluntary + IAP + colitis	652 ± 10 *
*HFD*	Seden	456 ± 11 *^+^
	Seden + colitis	375 ± 15 ^+^
	Voluntary + colitis	576 ± 17 ^++^
	Voluntary + IAP + colitis	624 ± 14 ^+++^

## Data Availability

All data is contained within the article.
